# {[1-(2-Amino­ethyl­amino)-1-methyl­ethyl]phospho­nato-κ^3^ *N*,*N*′,*O*}chloridopalladium(II) monohydrate

**DOI:** 10.1107/S1600536810001765

**Published:** 2010-01-20

**Authors:** Anatolij Dudko, Vladimir Bon, Alexandra Kozachkova, Natalia Tsaryk, Vasily Pekhnyo

**Affiliations:** a Institute of General and Inorganic Chemistry, NAS Ukraine, Kyiv, prosp. Palladina 32/34, 03680 Ukraine

## Abstract

In the title compound, [Pd(C_5_H_14_N_2_O_3_P)Cl]·H_2_O, the Pd(II) atom shows a slightly distorted square-planar geometry and forms two five-membered metallacycles, which both exhibit half-chair conformations. The crystal structure consists of layers propogating in the [100] direction which are connected into a three-dimensional network by strong N—H⋯Cl, N—H⋯O and O—H⋯O hydrogen bonds.

## Related literature

For general background to the use of organic phospho­nic acids as chelating agents in metal extraction and as drugs for the prevention of calcification and bone resorption, see: Matczak-Jon & Videnova-Adrabinska (2005 ▶); Tromelin *et al.* (1986 ▶); Szabo *et al.* (2002 ▶). For related structures, see: Shkol’nikova *et al.* (1991 ▶).
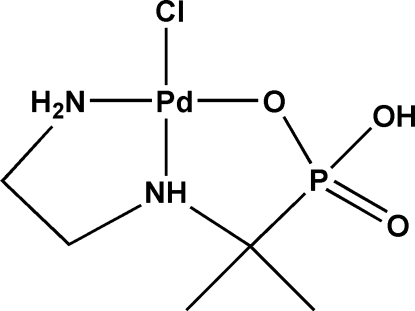


## Experimental

### 

#### Crystal data

[Pd(C_5_H_14_N_2_O_3_P)Cl]·H_2_O
*M* *_r_* = 341.02
Triclinic, 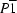

*a* = 7.2158 (2) Å
*b* = 7.8981 (2) Å
*c* = 10.3179 (3) Å
α = 97.968 (2)°
β = 98.403 (2)°
γ = 95.894 (2)°
*V* = 571.55 (3) Å^3^
*Z* = 2
Mo *K*α radiation
μ = 1.99 mm^−1^
*T* = 100 K
0.38 × 0.12 × 0.10 mm

#### Data collection

Bruker APEXII CCD diffractometer
Absorption correction: multi-scan (*SADABS*; Bruker, 2005 ▶) *T*_min_ = 0.519, *T*_max_ = 0.832
8452 measured reflections
2306 independent reflections
1954 reflections with *I* > 2σ(*I*)
*R*_int_ = 0.046

#### Refinement

*R*[*F* ^2^ > 2σ(*F* ^2^)] = 0.032
*wR*(*F* ^2^) = 0.076
*S* = 1.05
2306 reflections
147 parameters
1 restraint
H atoms treated by a mixture of independent and constrained refinement
Δρ_max_ = 0.75 e Å^−3^
Δρ_min_ = −0.55 e Å^−3^

### 

Data collection: *APEX2* (Bruker, 2005 ▶); cell refinement: *SAINT* (Bruker, 2005 ▶); data reduction: *SAINT*; program(s) used to solve structure: *SHELXS97* (Sheldrick, 2008 ▶); program(s) used to refine structure: *SHELXL97* (Sheldrick, 2008 ▶); molecular graphics: *SHELXTL* (Sheldrick, 2008 ▶); software used to prepare material for publication: *publCIF* (Westrip, 2010 ▶).

## Supplementary Material

Crystal structure: contains datablocks I, global. DOI: 10.1107/S1600536810001765/im2173sup1.cif

Structure factors: contains datablocks I. DOI: 10.1107/S1600536810001765/im2173Isup2.hkl

Additional supplementary materials:  crystallographic information; 3D view; checkCIF report

## Figures and Tables

**Table 1:** Hydrogen-bond geometry (Å, °)

*D*—H⋯*A*	*D*—H	H⋯*A*	*D*⋯*A*	*D*—H⋯*A*
N1—H1*N*⋯Cl1^i^	0.86 (5)	2.48 (5)	3.326 (4)	169 (4)
N2—H21*N*⋯O3^i^	0.96 (5)	1.98 (5)	2.937 (5)	177 (4)
N2—H22*N*⋯Cl1^ii^	0.76 (5)	2.68 (5)	3.365 (4)	151 (4)
O3—H3*O*⋯O2^iii^	0.77 (3)	1.75 (3)	2.509 (4)	168 (6)
O4—H41*O*⋯O2^iv^	0.79 (5)	2.14 (6)	2.911 (5)	166 (5)
O4—H42*O*⋯O1	0.79 (6)	2.08 (6)	2.854 (5)	167 (5)

Symmetry codes: (i) 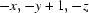; (ii) 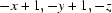; (iii) 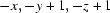; (iv) 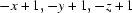.

**Fig. 1.**
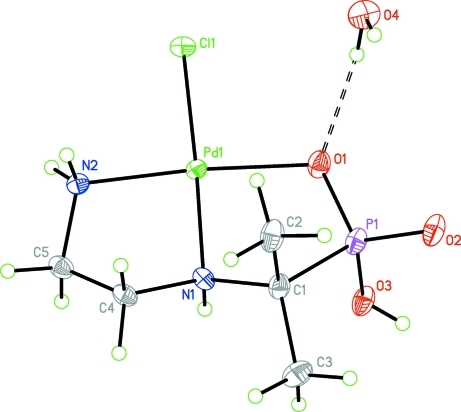
The title compound showing 50% probability displacement ellipsoids for non-hydrogen atoms.

**Fig. 2.**
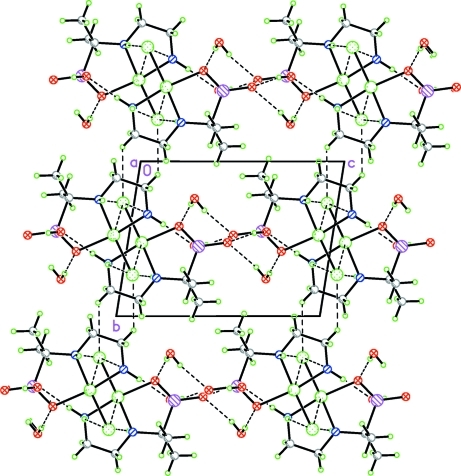
Crystal packing of the title compound, projection down the a axis. Dashed lines indicate hydrogen bonds.

## supplementary crystallographic information

### Comment

Organic phosphonic acids are potentially very powerful chelating agents used in metal extractions and they are also tested by pharmaceutical industry for use as efficient drugs preventing calcification and inhibiting bone resorption (Tromelin *et al.*, 1986, Matczak-Jon & Videnova-Adrabinska, 2005). Diphosphonic acids are used in the treatment of Paget disease, osteoporosis and tumoral osteolysis (Szabo *et al.*, 2002). The molecular structure of the title compound contains one molecule of the complex per asymmetric unit (Fig.1). The palladium atom shows a slightly distorted square-planar geometry. Mean average deviation from the respective plane is 0.040 (1) Å with a maximum deviation for O1 of 0.048 (1) Å. The bond lengths have a good correlation with reference data (Shkol'nikova *et al.*, 1991). The ligand molecule coordinated to the palladium atom in a tridentate manner *via* phosphonic oxygen and two amino nitrogen atoms creating two five-membered metallacyclic subunits in half-chair conformation. Torsion angles C1–P1–O1–Pd1 = -26.4 (2)° and Pd1–N1–C1–P1 = -43.9 (3)° of the metallacycle [Pd1O1P1C1N1] slightly differ from the corresponding angles Pd1–N1–C4–C5 = 42.4 (4)° and Pd1–N2–C5–C4 = 37.5 (4)° of the second metallacycle [PdN1C4C5N2] because of different stereochemical environments. The crystal structure of the title compound forms a layered supramolecular structure, stabilized by strong N–H···Cl, N–H···O and O–H···O hydrogen bonds (Fig.2, Table 1).

### Experimental

2-(2-aminoethyl)aminopropan-2-yl-phosphonic acid hydrochloride (0.219 g, 1 mmol) in water (10 ml) was mixed together with a solution of palladium diacetate (0.224 g, 1 mmol, Merck ≥ 99%) in benzene (10 ml). The color of the aqueous phase of the reaction mixture slowly turned to pale yellow. After stirring for 12 h, the aqueous phase of the solution was separated. Suitable single crystals of the title compound were produced by slow evaporation of water from an aqueous solution at room temperature (yield: 76%). A pale yellow needle-shaped crystal was used for data collection.

### Refinement

H atoms bonded to N and O atoms were located in a difference map and refined with constrained *U*_iso_(H) = 1.2*U*_eq_(N,*O*). Other H atoms were positioned geometrically and refined using riding model with C–H = 0.99 Å for CH_2_ [*U*_iso_(H) = 1.2Ueq(C)] and C–H = 0.98 Å for CH_3_ [*U*_iso_(H) = 1.5*U*_eq_(C)]. The *DFIX* instruction was used in the final refinement for restraining the O3—H3O distance to a reasonable value.

### Crystal data

**Table:**

[Pd(C_5_H_14_N_2_O_3_P)Cl]·H_2_O	*Z* = 2
*M**_r_* = 341.02	*F*(000) = 340
Triclinic, *P*1	*D*_x_ = 1.982 Mg m^−^^3^
Hall symbol: -P 1	Melting point: 535 K
*a* = 7.2158 (2) Å	Mo *K*α radiation, λ = 0.71073 Å
*b* = 7.8981 (2) Å	Cell parameters from 2725 reflections
*c* = 10.3179 (3) Å	θ = 2.9–26.2°
α = 97.968 (2)°	µ = 1.99 mm^−^^1^
β = 98.403 (2)°	*T* = 100 K
γ = 95.894 (2)°	Block, yellow
*V* = 571.55 (3) Å^3^	0.38 × 0.12 × 0.10 mm

### Data collection

**Table:**

Bruker APEXII CCD diffractometer	2306 independent reflections
Radiation source: fine-focus sealed tube	1954 reflections with *I* > 2σ(*I*)
graphite	*R*_int_ = 0.046
Detector resolution: 8.26 pixels mm^-1^	θ_max_ = 26.4°, θ_min_ = 2.0°
φ and ω scans	*h* = −8→8
Absorption correction: multi-scan (*SADABS*; Bruker, 2005)	*k* = −9→9
*T*_min_ = 0.519, *T*_max_ = 0.832	*l* = −12→12
8452 measured reflections	

### Refinement

**Table:**

Refinement on *F*^2^	Primary atom site location: structure-invariant direct methods
Least-squares matrix: full	Secondary atom site location: difference Fourier map
*R*[*F*^2^ > 2σ(*F*^2^)] = 0.032	Hydrogen site location: inferred from neighbouring sites
*wR*(*F*^2^) = 0.076	H atoms treated by a mixture of independent and constrained refinement
*S* = 1.05	*w* = 1/[σ^2^(*F*_o_^2^) + (0.0351*P*)^2^ + 0.6204*P*] where *P* = (*F*_o_^2^ + 2*F*_c_^2^)/3
2306 reflections	(Δ/σ)_max_ < 0.001
147 parameters	Δρ_max_ = 0.75 e Å^−^^3^
1 restraint	Δρ_min_ = −0.55 e Å^−^^3^

### Special details

**Table:**

Geometry. All e.s.d.'s (except the e.s.d. in the dihedral angle between two l.s. planes) are estimated using the full covariance matrix. The cell e.s.d.'s are taken into account individually in the estimation of e.s.d.'s in distances, angles and torsion angles; correlations between e.s.d.'s in cell parameters are only used when they are defined by crystal symmetry. An approximate (isotropic) treatment of cell e.s.d.'s is used for estimating e.s.d.'s involving l.s. planes.
Refinement. Refinement of *F*^2^ against ALL reflections. The weighted *R*-factor *wR* and goodness of fit *S* are based on *F*^2^, conventional *R*-factors *R* are based on *F*, with *F* set to zero for negative *F*^2^. The threshold expression of *F*^2^ > σ(*F*^2^) is used only for calculating *R*-factors(gt) *etc*. and is not relevant to the choice of reflections for refinement. *R*-factors based on *F*^2^ are statistically about twice as large as those based on *F*, and *R*- factors based on ALL data will be even larger.

### Fractional atomic coordinates and isotropic or equivalent isotropic displacement parameters (Å^2^)

**Table:**

	*x*	*y*	*z*	*U*_iso_*/*U*_eq_	
Pd1	0.22708 (4)	0.52397 (4)	0.07010 (3)	0.01203 (11)	
P1	0.15753 (15)	0.54887 (16)	0.35047 (10)	0.0162 (3)	
Cl1	0.27656 (14)	0.26896 (14)	−0.05111 (10)	0.0155 (2)	
N1	0.1808 (5)	0.7519 (4)	0.1696 (3)	0.0120 (7)	
H1N	0.060 (7)	0.746 (6)	0.150 (4)	0.014*	
N2	0.2614 (5)	0.6578 (5)	−0.0782 (3)	0.0126 (7)	
H21N	0.199 (6)	0.594 (6)	−0.161 (4)	0.015*	
H22N	0.368 (7)	0.665 (6)	−0.078 (4)	0.015*	
O1	0.2068 (4)	0.4194 (4)	0.2400 (3)	0.0184 (7)	
O2	0.2368 (4)	0.5237 (4)	0.4882 (3)	0.0209 (7)	
O3	−0.0616 (4)	0.5451 (4)	0.3289 (3)	0.0190 (7)	
H3O	−0.117 (6)	0.538 (7)	0.387 (4)	0.023*	
O4	0.5268 (5)	0.2409 (5)	0.2944 (3)	0.0249 (8)	
H41O	0.577 (8)	0.316 (7)	0.351 (5)	0.030*	
H42O	0.428 (8)	0.276 (7)	0.281 (5)	0.030*	
C1	0.2460 (6)	0.7633 (6)	0.3169 (4)	0.0158 (9)	
C2	0.4630 (6)	0.7880 (6)	0.3480 (4)	0.0214 (10)	
H2A	0.5114	0.9015	0.3301	0.032*	
H2B	0.5122	0.6982	0.2921	0.032*	
H2C	0.5036	0.7801	0.4416	0.032*	
C3	0.1618 (7)	0.9064 (6)	0.3944 (4)	0.0237 (10)	
H3A	0.2125	1.0182	0.3746	0.036*	
H3B	0.1944	0.9037	0.4896	0.036*	
H3C	0.0241	0.8895	0.3688	0.036*	
C4	0.2573 (6)	0.8907 (5)	0.1007 (4)	0.0157 (9)	
H4A	0.3965	0.9126	0.1242	0.019*	
H4B	0.2044	0.9989	0.1265	0.019*	
C5	0.2000 (6)	0.8285 (6)	−0.0464 (4)	0.0168 (9)	
H5A	0.0613	0.8209	−0.0712	0.020*	
H5B	0.2596	0.9106	−0.0970	0.020*	

### Atomic displacement parameters (Å^2^)

**Table:**

	*U*^11^	*U*^22^	*U*^33^	*U*^12^	*U*^13^	*U*^23^
Pd1	0.01212 (17)	0.01164 (19)	0.01225 (17)	0.00112 (12)	0.00298 (11)	0.00076 (12)
P1	0.0142 (5)	0.0219 (7)	0.0118 (5)	−0.0005 (5)	0.0022 (4)	0.0023 (5)
Cl1	0.0142 (5)	0.0136 (6)	0.0183 (5)	0.0028 (4)	0.0027 (4)	0.0002 (4)
N1	0.0102 (17)	0.0102 (19)	0.0140 (17)	−0.0012 (15)	−0.0016 (13)	0.0022 (14)
N2	0.0097 (17)	0.013 (2)	0.0143 (18)	0.0005 (15)	0.0019 (14)	0.0013 (15)
O1	0.0237 (16)	0.0159 (17)	0.0148 (15)	−0.0016 (13)	0.0046 (12)	0.0013 (13)
O2	0.0154 (15)	0.033 (2)	0.0141 (15)	−0.0006 (14)	0.0034 (12)	0.0062 (14)
O3	0.0129 (15)	0.0294 (19)	0.0147 (15)	−0.0011 (14)	0.0034 (11)	0.0047 (14)
O4	0.0216 (18)	0.027 (2)	0.0244 (18)	0.0028 (16)	0.0009 (14)	−0.0003 (15)
C1	0.018 (2)	0.016 (2)	0.012 (2)	0.0002 (18)	0.0018 (16)	0.0017 (17)
C2	0.020 (2)	0.028 (3)	0.014 (2)	−0.003 (2)	0.0005 (17)	0.004 (2)
C3	0.027 (3)	0.023 (3)	0.019 (2)	0.003 (2)	0.0066 (19)	−0.004 (2)
C4	0.018 (2)	0.009 (2)	0.020 (2)	0.0005 (18)	0.0020 (17)	0.0024 (18)
C5	0.013 (2)	0.014 (2)	0.024 (2)	0.0019 (18)	0.0033 (17)	0.0062 (19)

### Geometric parameters (Å, °)

**Table:**

Pd1—N2	2.006 (3)	O4—H41O	0.79 (5)
Pd1—N1	2.029 (3)	O4—H42O	0.79 (6)
Pd1—O1	2.056 (3)	C1—C3	1.523 (6)
Pd1—Cl1	2.3083 (11)	C1—C2	1.538 (6)
P1—O2	1.500 (3)	C2—H2A	0.9800
P1—O1	1.530 (3)	C2—H2B	0.9800
P1—O3	1.561 (3)	C2—H2C	0.9800
P1—C1	1.844 (4)	C3—H3A	0.9800
N1—C4	1.490 (5)	C3—H3B	0.9800
N1—C1	1.511 (5)	C3—H3C	0.9800
N1—H1N	0.86 (5)	C4—C5	1.511 (6)
N2—C5	1.471 (5)	C4—H4A	0.9900
N2—H21N	0.96 (5)	C4—H4B	0.9900
N2—H22N	0.76 (5)	C5—H5A	0.9900
O3—H3O	0.77 (3)	C5—H5B	0.9900
			
N2—Pd1—N1	84.95 (14)	C3—C1—C2	111.8 (4)
N2—Pd1—O1	171.76 (13)	N1—C1—P1	103.1 (3)
N1—Pd1—O1	87.95 (12)	C3—C1—P1	111.8 (3)
N2—Pd1—Cl1	92.89 (11)	C2—C1—P1	108.9 (3)
N1—Pd1—Cl1	177.67 (10)	C1—C2—H2A	109.5
O1—Pd1—Cl1	94.26 (9)	C1—C2—H2B	109.5
O2—P1—O1	114.59 (18)	H2A—C2—H2B	109.5
O2—P1—O3	112.56 (16)	C1—C2—H2C	109.5
O1—P1—O3	107.83 (17)	H2A—C2—H2C	109.5
O2—P1—C1	111.12 (19)	H2B—C2—H2C	109.5
O1—P1—C1	105.66 (18)	C1—C3—H3A	109.5
O3—P1—C1	104.36 (19)	C1—C3—H3B	109.5
C4—N1—C1	118.5 (3)	H3A—C3—H3B	109.5
C4—N1—Pd1	107.3 (2)	C1—C3—H3C	109.5
C1—N1—Pd1	110.9 (3)	H3A—C3—H3C	109.5
C4—N1—H1N	105 (3)	H3B—C3—H3C	109.5
C1—N1—H1N	112 (3)	N1—C4—C5	106.8 (3)
Pd1—N1—H1N	101 (3)	N1—C4—H4A	110.4
C5—N2—Pd1	108.9 (2)	C5—C4—H4A	110.4
C5—N2—H21N	114 (3)	N1—C4—H4B	110.4
Pd1—N2—H21N	111 (3)	C5—C4—H4B	110.4
C5—N2—H22N	111 (4)	H4A—C4—H4B	108.6
Pd1—N2—H22N	103 (3)	N2—C5—C4	108.7 (3)
H21N—N2—H22N	109 (4)	N2—C5—H5A	109.9
P1—O1—Pd1	112.20 (17)	C4—C5—H5A	109.9
P1—O3—H3O	121 (4)	N2—C5—H5B	109.9
H41O—O4—H42O	97 (5)	C4—C5—H5B	109.9
N1—C1—C3	110.7 (3)	H5A—C5—H5B	108.3
N1—C1—C2	110.2 (3)		
			
N2—Pd1—N1—C4	−18.0 (3)	C4—N1—C1—P1	−168.6 (3)
O1—Pd1—N1—C4	157.8 (3)	Pd1—N1—C1—P1	−43.9 (3)
N2—Pd1—N1—C1	−148.9 (3)	O2—P1—C1—N1	170.2 (2)
O1—Pd1—N1—C1	26.9 (3)	O1—P1—C1—N1	45.4 (3)
N1—Pd1—N2—C5	−10.8 (3)	O3—P1—C1—N1	−68.2 (3)
Cl1—Pd1—N2—C5	168.3 (3)	O2—P1—C1—C3	−70.8 (3)
O2—P1—O1—Pd1	−149.06 (16)	O1—P1—C1—C3	164.3 (3)
O3—P1—O1—Pd1	84.75 (19)	O3—P1—C1—C3	50.7 (3)
C1—P1—O1—Pd1	−26.4 (2)	O2—P1—C1—C2	53.2 (3)
N1—Pd1—O1—P1	3.43 (18)	O1—P1—C1—C2	−71.6 (3)
Cl1—Pd1—O1—P1	−175.86 (15)	O3—P1—C1—C2	174.8 (3)
C4—N1—C1—C3	71.7 (5)	C1—N1—C4—C5	168.9 (3)
Pd1—N1—C1—C3	−163.6 (3)	Pd1—N1—C4—C5	42.4 (4)
C4—N1—C1—C2	−52.6 (5)	Pd1—N2—C5—C4	37.5 (4)
Pd1—N1—C1—C2	72.2 (4)	N1—C4—C5—N2	−53.3 (4)

### Hydrogen-bond geometry (Å, °)

**Table:**

*D*—H···*A*	*D*—H	H···*A*	*D*···*A*	*D*—H···*A*
N1—H1N···Cl1^i^	0.86 (5)	2.48 (5)	3.326 (4)	169 (4)
N2—H21N···O3^i^	0.96 (5)	1.98 (5)	2.937 (5)	177 (4)
N2—H22N···Cl1^ii^	0.76 (5)	2.68 (5)	3.365 (4)	151 (4)
O3—H3O···O2^iii^	0.77 (3)	1.75 (3)	2.509 (4)	168 (6)
O4—H41O···O2^iv^	0.79 (5)	2.14 (6)	2.911 (5)	166 (5)
O4—H42O···O1	0.79 (6)	2.08 (6)	2.854 (5)	167 (5)

Symmetry codes: (i) −*x*, −*y*+1, −*z*; (ii) −*x*+1, −*y*+1, −*z*; (iii) −*x*, −*y*+1, −*z*+1; (iv) −*x*+1, −*y*+1, −*z*+1.
